# Does preterm birth increase the initiation of antidepressant use during the postpartum? A population-based investigation

**DOI:** 10.3389/fphar.2024.1325381

**Published:** 2024-03-27

**Authors:** Giovanna Esposito, Anna Cantarutti, Angela Lupattelli, Matteo Franchi, Giovanni Corrao, Fabio Parazzini

**Affiliations:** ^1^ Department of Clinical Sciences and Community Health, University of Milan, Milan, Italy; ^2^ Unit of Biostatistics, Epidemiology and Public Health, Department of Statistics and Quantitative Methods, University of Milano-Bicocca, Milan, Italy; ^3^ National Centre for Healthcare Research and Pharmacoepidemiology, Milan, Italy; ^4^ PharmacoEpidemiology and Drug Safety Research Group, Department of Pharmacy, and PharmaTox Strategic Research Initiative, University of Oslo, Oslo, Norway

**Keywords:** preterm, antidepressant, depression, maternal mental health, postpartum

## Abstract

**Background:**

Preterm birth may affect maternal mental health. We explored the relationship between preterm birth and the risk of initiating antidepressant use during the year after birth.

**Methods:**

We conducted a population-based investigation using regional healthcare utilization databases. The exposure considered was preterm birth. The outcome was having at least one prescription for antidepressant medications during the year after birth. We used a log-binomial regression model including terms for maternal age at birth, nationality, educational level, parity, modality of conception, modality of delivery, use of other psychotropic drugs, and diabetes to estimate relative risk (RR) and 95% confidence intervals (CI) for the association between preterm birth and the initiation of antidepressant use. In addition, the absolute risk differences (ARD) were also computed according to the timing of birth.

**Results:**

The cohort included 727,701 deliveries between 2010 and 2020 in Lombardy, Northern Italy. Out of these, 6,522 (0.9%) women had at least one prescription for antidepressant drugs during the year after birth. Preterm births were related to a 38% increased risk of initiation of antidepressant use during the year after birth (adjusted RR = 1.38; 95% CI: 1.25–1.52) for moderate to late preterm and to 83% (adjusted RR = 1.83; 95% CI: 1.46–2.28) for extremely and very preterm. Excluding women with only one antidepressant prescription, the association was consistent (adjusted RR = 1.41, 95%CI: 1.23–1.61 for moderate to late preterm and adjusted RR = 1.81, 95% CI: 1.31–2.49 for extremely and very preterm). Also, excluding women who used other psychotropics, the association remained consistent (adjusted RR = 1.39, 95%CI: 1.26–1.54 and adjusted RR = 1.91, 95% CI: 1.53–2.38, respectively for moderate to late and extremely and very preterm).

**Conclusion:**

Women who delivered preterm may have an excess risk of initiation of antidepressant consumption during the first year after birth.

## 1 Introduction

Postpartum mental disorders, most commonly unipolar depression and anxiety, are important clinical and public health concerns. They can negatively affect women’s somatic and psychiatric health and ability to work; at worst, these disorders are associated with an increased risk of suicide (Chin et al., 2022). In addition, infants of mothers with postpartum depression have a greater risk for adverse developmental outcomes and poorer mother-child bonding (Parsons et al., 2012; Hoffman et al., 2017).

Mothers can experience mental and emotional disorders of various nature and severity in the postpartum period. The emotional and hormonal changes that accompany childbirth can affect mothers’ mood. More than one in three women experience the so-called “maternity blues”, and more rarely, severe and prolonged depression (Rezaie-Keikhaie et al., 2020; Tosto et al., 2023). The prevalence of postpartum depression is around 15%–20%, with some variability across populations (Zhao and Zhang, 2020; Liu et al., 2022). The depressive state, typically within the first year since childbirth (Hacking, 2013), comprises a wide range of symptoms such as difficulty bonding with the child, fear of not being a good mother, feelings of worthlessness, shame, guilt or inadequacy, and thoughts of harming oneself or the infant. Postpartum depression often co-exists with anxiety disorders, adding substantial burden to women’s mental health (Falah-Hassani et al., 2017). Postpartum psychosis, a life-threatening psychiatric emergency including acute mania or depression with psychosis, is infrequent and affects 1–2 women per 1,000 (Perry et al., 2021).

Antidepressant pharmacotherapy is indicated in situations characterized by moderate to severe severity or inadequate response to initial psychotherapy (Hendrick, 2006). Selective serotonin reuptake inhibitors are the mainstay of pharmacological treatment for perinatal depression, with an international prescribing prevalence of 4.7% in the first year postpartum (Molenaar et al., 2020). The management of antidepressant treatment in the postpartum period, particularly during the breastfeeding period, is a complex issue. The potential adverse effects of poorly managed peripartum maternal depression on both maternal and infant health must be balanced against the potential risks of drug exposure to the infant through breast milk. However, there is evidence that most antidepressants are not contraindicated during breastfeeding (Gentile, 2005; Kendall-Tackett and Hale, 2010; Stewart and Vigod, 2019). Even to date, many European countries lack consistent and up-to-date clinical practice guidelines for the pharmacological treatment of peripartum depression (Kittel-Schneider et al., 2022).

Environmental and socioeconomic factors, as well as psychiatric and obstetric conditions such as depression during pregnancy, history of depression, caesarean section, and gestational diabetes, play a crucial role in the incidence of postpartum depression (Ghaedrahmati et al., 2017; Zhao and Zhang, 2020; Liu et al., 2022). The role of preterm birth, the leading cause of neonatal deaths worldwide, has been previously explored, providing some evidence for an increased risk for postpartum depression, but results remain inconsistent (Vigod et al., 2010). Given the relevant impact of mental illness on mothers, infants, and families and the implications of preterm birth, it is of utmost importance to further investigate the association between the two conditions.

We conducted a population-based cohort study using the regional healthcare utilization databases in the Lombardy region of Italy to evaluate the potential association between preterm birth and maternal initiation of antidepressant use during the postpartum year, taking into account potential confounders, such as socio-demographic features and selected maternal clinical factors.

## 2 Materials and methods

### 2.1 Data source and cohort selection

Data for the study were obtained from the regional health databases of Lombardy, which collect a wide range of information on services provided to beneficiaries of the National Health Service (NHS), a tax-funded universal healthcare system that provides free or subsidized health services to all Italian citizens and residents, ensuring equal access for the entire population. The databases include the registry of diagnosis at discharge from public or private hospitals, the outpatient drug prescriptions reimbursed by the NHS registry, and the outpatient services registry. In addition, a specific form for the birth event, the certificate of delivery assistance (CedAP), reports information about maternal characteristics, pregnancy, and delivery. We linked the above databases using the unique identification code, which identified each selected unit across all databases, through a deterministic record linkage. For privacy issues, each identification code is automatically anonymized. This record linkage process offers the opportunity to design investigations including very large, unselected birth cohort populations and to generate real-world evidence on several fields of public health, including the birth event.

Deliveries that occurred in the region from 1st January 2010 to 1st January 2020 were identified. We excluded (1) deliveries that did not match a hospitalization related to childbirth, (2) deliveries of women that the NHS did not cover in the year before and after the birth, (3) deliveries of women aged less than 15 or more than 55, (4) deliveries before the 22nd or after the 42nd week of gestation, (5) stillbirths, (6) multiple deliveries, (7) deliveries of children with congenital malformations, and (8) deliveries of women who were not free of depression or anxiety in the year before the conception or during pregnancy (at least one of following events, i.e., having an inpatient and/or outpatient diagnosis of depression and/or anxiety and/or at least one prescription with antidepressant medications) (Supplementary Table S1). A one-year wash-out period before conception seems a reasonable choice. Given that depression usually requires long-term treatment or access to specialized care, it is reasonable to assume that a woman is free of depression if she has not accessed such care during this period. This timeframe is a compromise between the various criteria used to exclude women with current depression in randomized clinical trials of antidepressant treatment for postpartum depression, ranging from 6 months to 2 years (Brown et al., 2021).

### 2.2 Exposure: preterm birth

The exposure considered was preterm birth, defined as any birth before 37 completed weeks of pregnancy (Lawn et al., 2010). This information was available in CedAP database. We categorized preterm birth as binary (i.e., yes if preterm before 37 complete weeks and no if at term) and on the basis of gestational weeks according to World Health Organization (i.e., extremely and very preterm before 32 weeks, moderate to late preterm between 32 and 36 weeks) (WHO, 2023). Extremely preterm (i.e., <28 gestational weeks) was not evaluated separately since they represent less than 5% of all births before 37 weeks.

### 2.3 Outcome: initiation of antidepressant use during the year after birth

Information on the outcome of interest was obtained from the outpatient drug prescription registry. The outcome was the initiation of antidepressant use during the year after birth (time of follow-up), defined as having filled at least a prescription for antidepressant medications (ATC code: N06A). We also considered the year after birth, splitting the time in the first 6 months (early) and 7–12 months (later). The reference population included all women who did not start taking antidepressants during the year after giving birth.

### 2.4 Covariates

Information on covariates that were used for confounding adjustment was obtained from the CedAP registry and the outpatient drug prescriptions registry. We considered several baseline maternal characteristics that may affect initiation with antidepressants during the year postpartum and prematurity as sufficient set of confounders: demographic variables [i.e., maternal age at birth (<30, 30–34, 35–39, >39 years), nationality (i.e., Italian or not based on birthplace), educational level (i.e., university, high school, middle or primary school)], pregnancy covariates [i.e., parity (nulliparous or multiparous women), mode of conception (natural or medically assisted)], and clinical variables (i.e., diabetes, defined as at least one prescription of an antidiabetic drug in the year before and during pregnancy considering both gestational and pre-existing diabetes, and prescription of other psychotropics in the same period). Missing data (regarding educational level and modality of delivery) was random and minimal, at less than 0.3%. Therefore, births with missing data were excluded from analyses.

### 2.5 Statistical analysis

We computed the initiation of antidepressant use rate per 100 according to selected maternal characteristics (i.e., age, nationality, educational level, parity, and diabetes) and information related to birth (i.e., mode of conception, mode and timing of delivery). Chi-squared test and the test for trend were used to compare initiators of antidepressant use and no-initiators among the selected covariates, appropriately.

The relative risk (RR) estimates of antidepressant initiation with 95% confidence interval (CI) were computed. The use of the robust variance estimator to account for correlations within women with multiple pregnancies did not change the CI considerably in the unadjusted analyses, so correlation structures were omitted from all analyses. Results are presented according to two levels of adjustment. The first unadjusted analysis examined the association between each covariate and the outcome. Further, we assessed the RR of antidepressant initiation according to gestational age at birth, considering all covariates as confounders. In addition, the absolute risk differences (ARD) with 95%CI were computed.

We conducted several sensitivity and subgroup analyses to evaluate the effect of outcome misclassification and effect modification by several covariates. (i) We redefined outcome as having filled at least 2 prescriptions of antidepressants; women who had only one antidepressant prescription in the postpartum year were excluded. (ii) We also considered the timing of initiation of antidepressant use, i.e., early initiation in the first 6 months after delivery and late initiation in the second 6 months after delivery. (iii) To ensure a more accurate assessment of women’s mental health before pregnancy, we only included women with at least 4 years of health coverage prior to conception. Consequently, we excluded women who had at least an inpatient or outpatient diagnosis of depression or anxiety and/or a prescription filled for antidepressant medications. Extending the wash-out period to 4 years allowed us to include a larger sample of women who were unlikely to be suffering from depression or anxiety at the time of conception. It should be noted that we could not exclude the possibility that a small number of these women should be considered as having depression/anxiety that was not in complete remission. According to the American Psychological Association (American Psychological Association, 2013), remission is defined as an extended period without significant symptoms (often 2 months). However, the line between remission and complete freedom from depression remains unclear, as there are no standardized timeframe thresholds to separate the two states (Furukawa et al., 2008; de Zwart et al., 2019). In addition, (iv) we performed a subgroup analysis among those deliveries that did not record any prescription of other psychotropics (ATC: N03A and N05A) in the year before conception and during pregnancy. Finally, (v) we carried out stratified analyses by maternal age, nationality, educational level, parity, and mode of conception.

We performed analysis using the Statistical Analysis System Software (version 9.4; SAS Institute, Cary, NC, USA).

## 3 Results

During the study period 2010–2020, we identified 727,701 deliveries (see Supplementary Figure S1 for the definition of the cohort), of which 689,261 (94.7%) were at term and 38,440 (5.3%) preterm. During the year after birth, 526 women with preterm birth (1.37 per 100 births) and 5,996 (0.87 per 100 births) with term birth had at least one filled antidepressant prescription. In Supplementary Material (Supplementary Table S2; Supplementary Figure S2), we reported patterns of prescribed antidepressants and other psychotropics according to preterm birth status. The average number of antidepressant prescriptions during the year after pregnancy was around 2.6 for both women who gave birth prematurely and those who gave birth at term (*p* = 0.782); 279 of women who initiated antidepressant had more than one concomitant prescription for different antidepressants. Women who started taking antidepressants were more likely to use other psychotropic drugs than women who did not start taking antidepressants (about 10% versus 1%).

Table 1 provides the rate per 100 and the crude RR of antidepressant initiation according to selected covariates. The initiation rate per 100 deliveries increased with the increase of age, ranging from 0.78 for women aged less than 30 to 1.18 for women aged more than 39 (p-trend <0.001), and the timing of birth. In the latter case, it ranged from 0.87 in term deliveries to 1.83 in very preterm ones (p-trend<0.001). Conversely, the rate was lower among not Italian women (*p* < 0.001) and in multiparous ones (*p* < 0.001). Accordingly, the risk was increased for increasing age (RR = 1.52, 95% CI: 1.39–1.66 for women aged 39 years or more versus women aged less than 30 years), lower educational level (RR = 1.20, 95% CI: 1.13–1.28 for women who attended middle/primary school versus women who attended university), medically assisted conception (RR = 1.29, 95% CI: 1.14–1.48), cesarean section (RR = 1.39, 95% CI: 1.32–1.47), and preterm birth (RR = 1.50, 95% CI: 1.37–1.66 for births between 32 and 36 weeks and RR = 2.10, 95% CI: 1.69–2.62 for deliveries before the 32 complete weeks). The risk was decreased in not Italian women (RR = 0.65, 95% CI: 0.61–0.69). No difference emerged for parity (RR = 0.97, 95% CI: 0.92–1.02) and diabetes (RR = 1.17, 95% CI: 0.97–1.40).

**TABLE 1:** Selected maternal characteristics according to initiation of antidepressant use in the year after birth. Lombardy, Italy, 2010–2020.

Maternal characteristics	Reference population, N = 721,179		Antidepressant initiators, N = 6,522		Risk per 100	*p*-value^a^	RR (95% CI)^b^
	n	%	*n*	%			
Maternal age						<0.001	
<30	200,463	27.8	1,573	24.1	0.78		Ref.
30–34	256,656	35.6	2,213	33.9	0.85		1.10 (1.03–1.17)
35–39	203,893	28.3	2,016	30.9	0.98		1.26 (1.18–1.34)
>39	60,167	8.3	720	11.0	1.18		1.52 (1.39–1.66)
Nationality						<0.001	
Italian	551,497	76.5	5,437	83.4	0.98		Ref.
Not Italian	169,682	23.5	1,085	16.6	0.64		0.65 (0.61–0.69)
Educational level*						<0.001	
University	235,144	32.7	1,921	29.5	0.81		Ref.
High school	314,829	43.8	2,923	44.9	0.92		1.14 (1.07–1.20)
Middle/primary school	168,680	23.5	1,659	25.5	0.97		1.20 (1.13–1.28)
Diabetes						0.093	
No	709,993	99.4	6,404	98.2	0.89		Ref.
Yes	11,186	1.6	118	1.8	1.04		1.17 (0.97.1.40)
Parity						0.187	
Nulliparae	351,792	48.8	3,235	49.6	0.91		Ref.
Multiparae	369,387	51.2	3,287	50.4	0.88		0.97 (0.92–1.02)
ART*						<0.001	
No	697,580	97.1	6,249	96.3	0.89		Ref.
Yes	20,746	2.9	242	3.7	1.15		1.29 (1.14–1.48)
Modality of delivery*						<0.001	
Vaginal	533,646	74.1	4,375	67.2	0.81		Ref.
Cesarean section	186,207	25.9	2,131	32.8	1.13		1.39 (1.32–1.47)
Timing of birth						<0.001	
≥37	683,265	94.7	5,996	91.9	0.87		Ref.
32–36	33,625	4.7	446	6.8	1.31		1.50 (1.37–1.66)
<32	4,289	0.6	80	1.2	1.83		2.10 (1.69–2.62)

^a^ Chi-square test or test for trend.

^b^ Estimated from log-binomial regression model; * sum did not reach the total due to missing data.

Table 2 shows the RR of antidepressant initiation according to the timing of birth, adjusted for selected confounders. Having moderate to late preterm birth was associated with a 38% increased risk of maternal antidepressant initiation during the year after birth (adjusted RR = 1.38; 95% CI: 1.25–1.52), relative to term birth. For extremely and very preterm births, there was a 83% increased risk (adjusted RR = 1.83; 1.46–2.28). In absolute terms, the ARD was 0.34% (95% CI: 0.22%–0.46%) for moderate to late preterm births and 0.85% (95%CI: 0.45%–1.24%).

**TABLE 2:** Relative risk (RR) and absolute risk difference (ARD) and corresponding 95% confidence interval (CI) of initiation of antidepressant use in the year after the birth according to gestational age at birth. Lombardy, Italy, 2010–2020.

Gestational age at birth (weeks)	Reference population, N (%)	Antidepressant initiators, N (%)	RR (95% CI)^a^	ARD (95% CI)^b^
Overall				
≥37	683,265 (94.7)	5,996 (91.9)	Ref.	Ref.
32–36	33,625 (4.7)	446 (6.8)	1.38 (1.25–1.52)	0.34 (0.22–0.46)
<32	4,289 (0.6)	80 (1.2)	1.83 (1.46–2.28)	0.85 (0.45–1.24)
Vaginal delivery				
≥37	514,570 (96.4)	4,164 (95.2)	Ref.	Ref.
32–36	17,756 (3.3)	189 (4.3)	1.29 (1.12–1.59)	0.24 (0.09–0.39)
<32	1,320 (0.3)	22 (0.5)	2.05 (1.35–3.10)	0.84 (0.16–1.52)
Cesarean section				
≥37	167,561 (90.0)	1,821 (85.4)	Ref.	Ref.
32–36	15,734 (8.5)	253 (11.9)	1.47 (1.29–1.67)	0.48 (0.28–0.67)
<32	2,912 (1.6)	57 (2.7)	1.77 (1.36–2.30)	0.88 (0.38–1.37)

^a^ Estimated from log-binomial regression model including terms for maternal age at birth, nationality, educational level, parity, modality of conception, mode of delivery, use of other psychotropics, and diabetes.

^b^ Adjusted for maternal age at birth, nationality, educational level, parity, modality of conception, mode of delivery, use of other psychotropics, and diabetes.

The results were confirmed in the sensitivity analysis, where we changed the outcome definition to requiring women to have filled at least 2 prescriptions within the year after childbirth (adjusted RR = 1.41, 95% CI: 1.23–1.61 in moderate to late preterm birth and adjusted RR = 1.81, 95% CI: 1.31–2.49 in extremely and very preterm births). In addition, when we divided the postpartum year into early and late, we observed a slightly weaker association with antidepressant initiation in later postpartum. For early antidepressant initiation, the adjusted RR was 1.43 (95% CI: 1.25–1.64) for moderate to late preterm births and 1.92 (95% CI: 1.42–2.61) for extreme and very preterm births. For later antidepressant initiation, the adjusted RR was 1.34 (95% CI: 1.16–1.54) and 1.75 (95% CI: 1.26–2.43) for moderate to late and for extremely and very preterm births, respectively.

When we excluded women without health coverage within the 4 years before the conceptions (N = 80,801) and those with at least (i) an inpatient and/or outpatient diagnosis of depression and/or anxiety, and/or (ii) at least one prescription with antidepressant medications (N = 24,017), the association between preterm birth and initiation of antidepressant use was consistent (adjusted RR = 1.37, 95% CI: 1.22–1.53 in moderate to late preterm birth and adjusted RR = 1.94, 95% CI: 1.50–2.50 in extremely and very preterm births). Therefore, we also estimated that only 3.3% of the women we defined as free of depression could be considered as having depression/anxiety in remission because they had been treated for or diagnosed with it in the 3 years prior to the year before conception. However, the main results were confirmed in sensitivity analyses where we changed the depression-free definition to women without antidepressant prescriptions and/or inpatient/outpatient diagnosis of depression/anxiety during the 4 years before conception.

In addition, considering the subgroup of those deliveries that did not record any prescription of other psychotropics in the year before the conception and during pregnancy, the association remained consistent, the adjusted RR was 1.39 (95%CI: 1.26–1.54) for moderate to late preterm births and 1.91 (95% CI: 1.53–2.38) for extremely and very preterm births.

**FIGURE 1:**
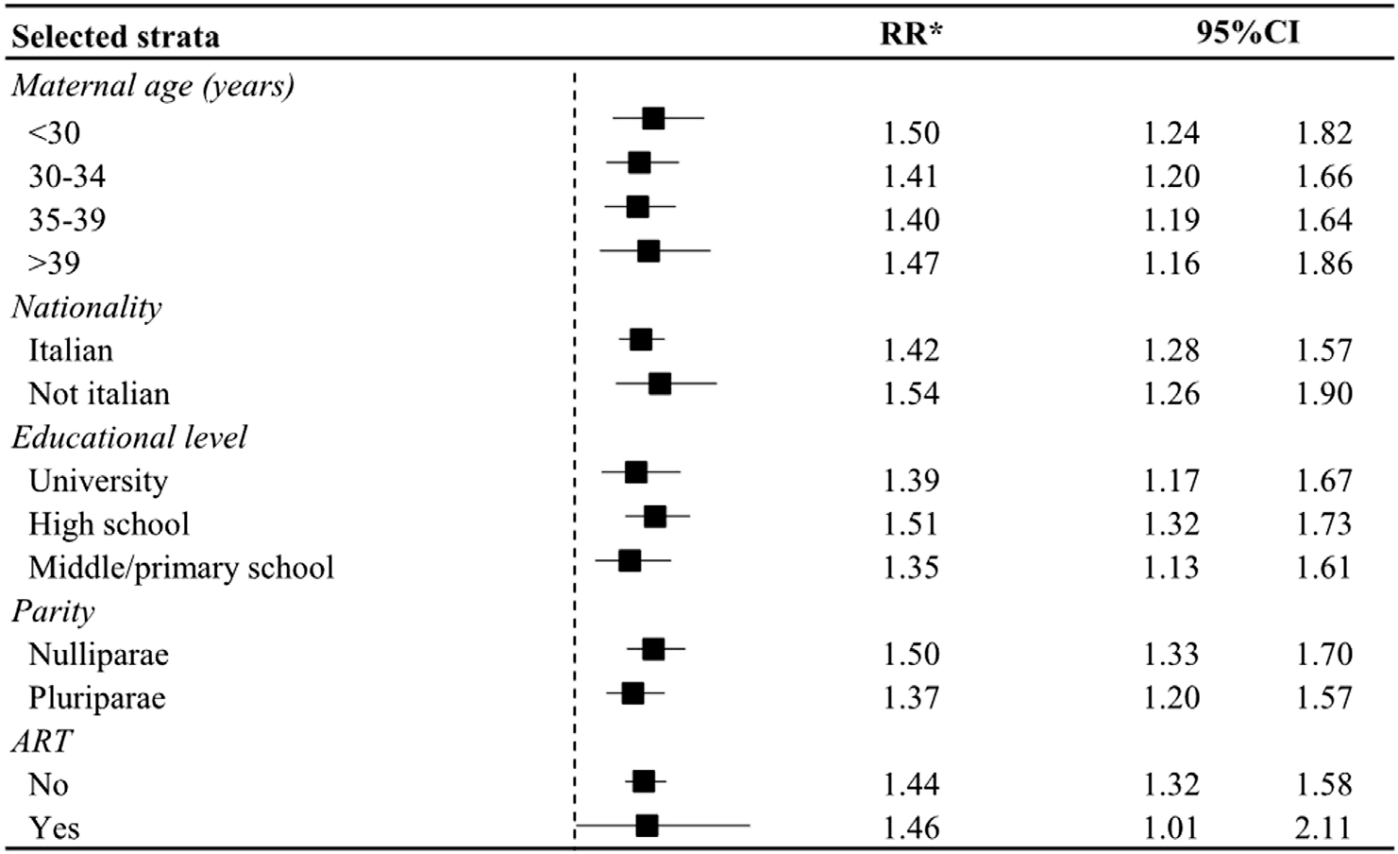
Relative Risk (RR) and corresponding 95% confidence interval (CI) of initiation of antidepressant use in the year after the birth in women who had a preterm birth in strata of selective covariates. Lombardy, Italy, 2010–2020. * Estimated from log-binomial regression model including terms for maternal age at birth, nationality, educational attainment, parity, modality of conception, mode of delivery, use of other psychotropics, and diabetes; unless the variable was the stratification factor.

There was no evidence of effect modification by the considered covariates. Indeed, results were consistent in the subgroup analyses performed (Figure 1).

## 4 Discussion

Our study found that among mothers free of depression for at least a year before conception, the risk of initiating antidepressant treatment during the first year after childbirth was 0.9%; mothers who experienced a moderate to late preterm birth had an additional risk of 0.3%, and those who experienced an extremely and very preterm birth had an additional risk of 0.9%. These estimates remained consistent with a more conservative outcome definition, requiring at least two filled prescriptions for antidepressant in the postpartum year.

Antidepressants, such as selective serotonin reuptake inhibitors, are the mainstay of pharmacological treatment for perinatal depression; fluvoxamine, paroxetine, and sertraline are preferred in breastfeeding women, leading to the lowest serum medication levels in infants (Langan and Goodbred, 2016). According to a study conducted in the USA, about half of women diagnosed with postpartum depression are on antidepressant treatment (Vu and Shaya, 2017). It is worth noting that treatment approaches for postpartum depression vary from region to region. For example, European countries tend to have lower levels of antidepressant use after childbirth than the USA (Molenaar et al., 2020). This variation between geographical areas may be influenced by cultural norms, differences in healthcare systems, and the availability and accessibility of alternative interventions. However, postpartum depression is not treated solely with antidepressants; the American Psychiatric Association and the National Institute for Health and Care Excellence recommend evidence-based treatments, including cognitive behavioural therapy and interpersonal psychotherapy, and suggest that non-medication psychotherapy should be considered as a potential first-line treatment for women with mild to moderate depression who are pregnant or breastfeeding (Gelenberg et al., 2010; NICE, 2020). It is reasonable to assume that in countries where the use of antidepressants is low, other options are considered. Having a preterm birth represents a stressful experience that could lead to depressive symptoms. According to a systematic review (Vigod et al., 2010), mothers of preterm infants were at high risk of depression in the immediate postpartum period in comparison to mothers of at term infants. In addition, mothers of very preterm birth have higher levels of depressive symptoms throughout the first postpartum year, with a limited reduction in symptoms after hospital discharge. However, not all the studies included provided consistent evidence. In light of these findings, it is imperative to emphasize the importance of implementing targeted interventions and support for mothers of preterm infants in the immediate postpartum period. These interventions may include providing comprehensive psychological support, facilitating access to mental health services, offering educational resources on coping strategies, and establishing community-based support groups tailored to the specific needs of preterm mothers. In addition, healthcare providers should prioritize regular screening for postpartum depression in this vulnerable population to ensure timely identification and intervention.

One plausible explanation of the relationship between preterm birth and the initiation of antidepressant after birth is the enhanced stress with increasing prematurity; the neonatal comorbidity, the length of hospitalization, and the difficulties in the management of the newborn are inversely proportional to gestational age (Vigod et al., 2010). Moreover, as premature infants may need to spend time in intensive care units, mothers of these babies experienced a limited opportunity to bond immediately after birth, which can hinder adequate attachment between mother and infant and affect maternal mood (Vigod et al., 2010). A study from Wales including parents with an infant admitted to a neonatal intensive care unit reported a dose-response relationship between the level of prematurity and depressive symptoms; there were higher depression scores in mothers of infants born before the 33rd week than in mothers of infants born at 33–35 weeks’ gestation and mothers of term infants (Carter et al., 2005). Our findings aligned with this hypothesis; the risk of maternal mental illness was higher in the subset of births before the 32nd week compared to the subset of births between 32 and 36 weeks. However, evidence was not fully consistent, a large prospective study conducted in 15 countries around the world found no association between the duration of parent-infant closeness during the first weeks of hospitalization and parental depressive symptoms (Lehtonen et al., 2022).

The temperament of preterm babies is less regulated and shows less attention and more signs of activity than babies born at term (Montirosso et al., 2016; Cassiano et al., 2020). The relationship between perceived difficult temperament in infants by mothers and maternal-infant attachment has been investigated as the mediating role of maternal health (Tolja et al., 2020; Davies et al., 2021; Li, 2022). Poor infant engagement and orientation, typical of preterm babies, has been shown to negatively affect the way in which a mother feels about her infant (Beebe et al., 2012). In a survey study of over 250 mothers of healthy term newborns during the first postpartum month, an association of difficult infant temperament with maternal postpartum anxiety and depressive symptoms emerged early in the postpartum period, independently of their known contributors to postpartum mood (Britton, 2011). The cause-and-effect relationship between maternal perinatal mood disorders and difficult infant behaviour could be considered bidirectional. As infant temperamental difficulty predisposes to altered maternal mood, poor mental health has been reported to impact the mother’s capacity for, and quality of, bonding and interaction with her child (Binda et al., 2019). This aspect represents a priority for public health since attachment between mother and newborn at the beginning of life significantly impacts a child’s wellbeing in later years, his emotional, social, and cognitive development.

Evidence suggests that sleep disturbance during pregnancy may increase the risk of postpartum depression (Maghami et al., 2021; Li et al., 2023). As sleep problems tend to last longer in mothers of preterm babies than in mothers of term babies (Toda Miyano et al., 2022), sleep disturbance may be another reason for the association between maternal depression and preterm birth. A preliminary study to assess the feasibility of a prospective, comparative, longitudinal study of the sleep and psychosocial health of parents of preterm and term infants during the first year after birth found that parents in the preterm group had a lower median total sleep time than parents in the term group, and that mothers in the preterm group tended to sleep more during the day than mothers in the term group (Marthinsen et al., 2022). This study has several strengths. First, this was a very large population-based cohort study covering all regional residents and a span of over 10 years. Second, the availability of high-quality integrated individual data from healthcare utilization databases on outpatient and inpatient services provided by the NHS and the record linkage process offers the opportunity to trace and evaluate the complete care pathway of women included. Our study also has several potential limitations. Most importantly, confounding variables, such as lifestyle factors (e.g., smoking, alcohol use, obesity), are unknown in administrative databases. This could result in residual confounding. Moreover, redeeming a prescription does not necessarily imply that the woman took the medication; we have no information available in this study to further examine this possibility (Corrao and Mancia, 2015). On the other hand, using data from outpatient drug prescriptions reimbursed by the NHS registry, we underestimated the actual use of antidepressants and the prevalence of postpartum depression. One reason could be that some drugs were prescribed outside the NHS system. However, there is no reason to believe that this proportion differed between at term and preterm births. The study may potentially undercount depression also using just pharmaceutical prescriptions. A systematic review and meta-analysis including 29 cohort studies and 4 case-control studies from different countries of the world reported a prevalence of postpartum depression of 14% (ranging from 5% to 26%); the presence of disease was assessed using the Edinburgh Postpartum Depression Scale in all included studies (Liu et al., 2022). In addition, the use of antidepressants in the years after childbirth can be a proxy for postpartum depression but also for other mental illnesses such as anxiety or even bulimia nervosa. Importantly, we must consider that women may opt not to take antidepressants specifically because they are pregnant or trying to conceive. Understanding that untreated depression during pregnancy carries risks for preterm birth (Jahan et al., 2021), we conducted a sensitivity analysis including only women with no signs of depression or anxiety for at least 4 years before conception, and our findings remained consistent.

In conclusion, our study sheds light on the relationship between the experience of preterm birth and the subsequent development of new-onset mental illness in the postpartum period, focusing on a population of healthy women free of depression/anxiety condition at least 1 year before the conception. These findings highlight the urgent need for evidence-based approaches to improve care and not neglect mental health in high risk childbirth conditions. Given the association we observed between the experience of preterm birth and antidepressant initiation, it is clear that strategies to ensure a healthy and full-term pregnancy are not only an important step in promoting maternal and fetal wellbeing during pregnancy, but also play a crucial role in reducing the risk of postpartum maternal mental illness. Looking forward, we might consider how longitudinal analysis could be crucial in deepening our understanding of the long-term effects of preterm birth on maternal mental health. Examining the persistence and development of depressive symptoms over time could provide valuable insights into the dynamic nature of this relationship. In addition, a longitudinal approach could help to identify any risk or protective factors that influence maternal mental health in the long term after preterm birth, thereby informing more effective intervention and support strategies.

## Data Availability

The original contributions presented in the study are included in the article/Supplementary Material, further inquiries can be directed to the corresponding author.
