# Supplementary material for: Does preterm birth increase the initiation of antidepressant use during the postpartum? A population-based investigation
Source: Front Pharmacol. 2024 Mar 27;15:1325381. doi: 10.3389/fphar.2024.1325381 (PMC11004433; doi:10.3389/fphar.2024.1325381)
Supplement: Supplementary file 1 [file DataSheet1.pdf]

Table S1. Definition of hospital admission diagnoses and ATC codes used for the outcome measure and for the set of covariates measured.

**ICD-9- CM diagnostic codes**	**Description**
293.83, 293.84, 296.2, 296.3, 296.9, 298.0, 300.0, 300.02, 300.1, 300.2, 300.3, 300.4, 300.8, 300.9, 308.0, 309.0, 309.1, 309.24, 309.28, 309.81, 311, 313.0	Anxiety and depression
**ATC codes**	**Description**
A10	Antidiabetics
N06A	Antidepressants
N05A	Antipsychotics
N03A	Antiepileptic

Table S2. Type of antidepressants used according to timing of birth. Lombardy, Italy, 2010-2020.

Class	Drug	At term		Preterm	
		Number of prescriptions	% of users	Number of prescriptions	% of users
**Non-selective monoamine reuptake inhibitors**	Amitriptyline	608	10.1	63	12.0
	Clomipramine	38	0.6	4	0.8
	Trimipramine	13	0.2	1	0.2
	Nortriptyline	3	0.1	1	0.2
	Imipramine	2	0.0	0	0.0
**Selective serotonin reuptake inhibitors**	Paroxetine	1451	24.2	116	22.1
	Sertraline	1238	20.6	106	20.2
	Escitalopram	1233	20.6	107	20.3
	Citalopram	894	14.9	74	14.1
	Fluoxetine	239	4.0	21	4.0
	Fluvoxamine	57	1.0	7	1.3
**Other antidepressants**	Venlafaxine	388	6.5	26	4.9
	Duloxetine	222	3.7	18	3.4
	Mitrazapine	125	2.1	17	3.2
	Trazodone	95	1.6	17	3.2
	Vortioxetina	50	0.8	12	2.3
	Bupropion	24	0.4	3	0.6
	Mianserin	3	0.1	0	0.0
	Reboxetine	3	0.1	0	0.0

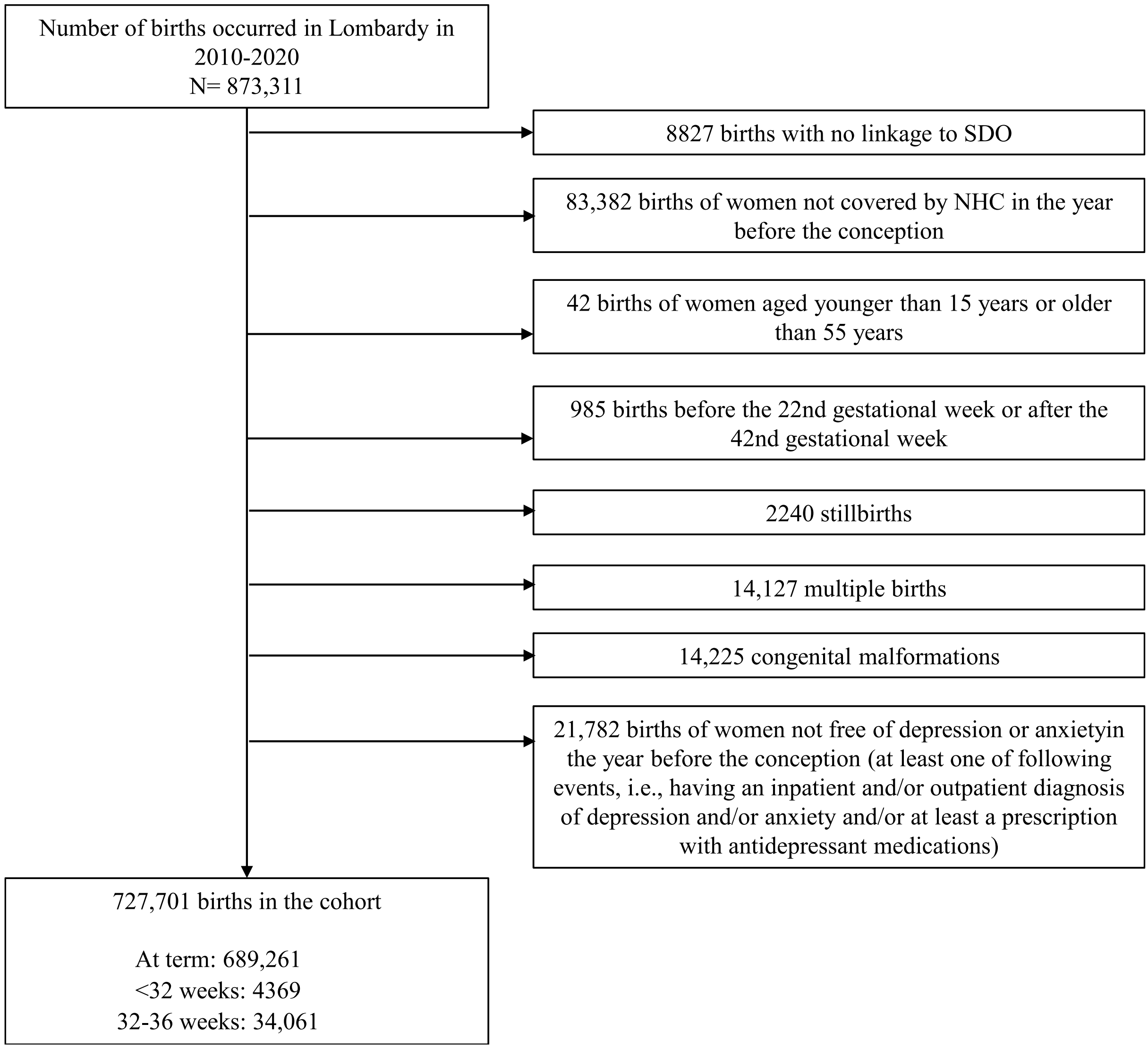

Figure S1. Flowchart of inclusion and exclusion criteria in the study cohort. Lombardy, Italy, 2010-2020.

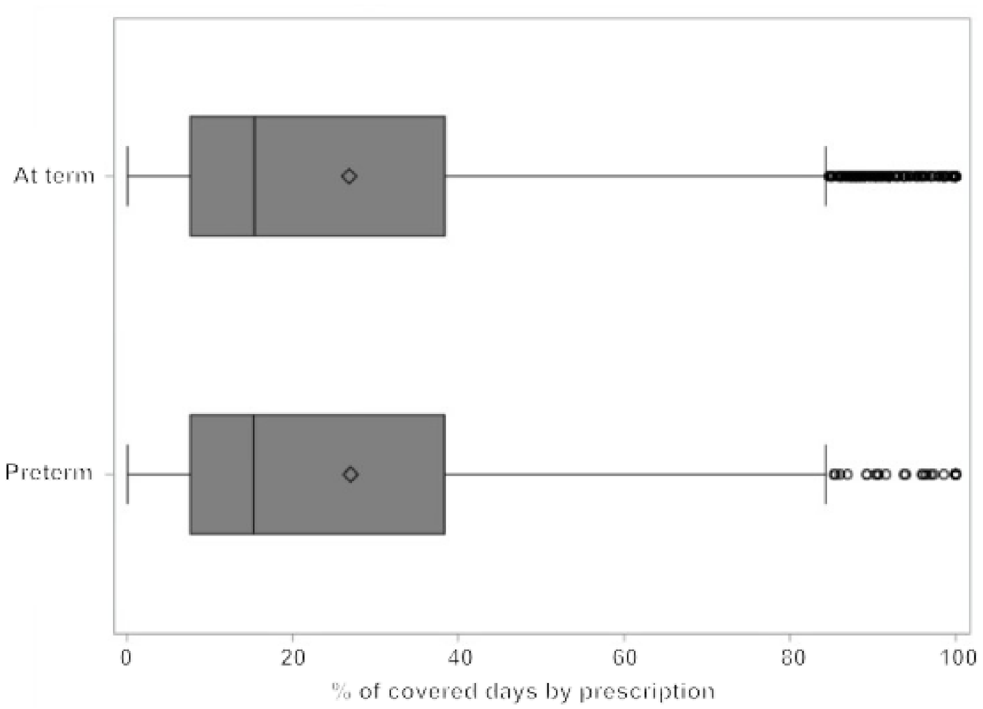

	At term, %	Preterm, %	Other psychotropic drugs: In the year before conception or during pregnancy	Other psychotropic drugs: In the year after birth
Initiators of antidepressant	89.3	87.3		
	8.1	10.5		
	1.6	1.7		
	1.0	0.6		
Reference population	99.2	98.8		
	0.4	0.4		
	0.3	0.4		
	0.2	0.3		

Figure S2. Patterns of prescribed antidepressants according to gestational age (panel A: % of covered days by prescription, panel B: use of other psychotropic drugs). Lombardy, Italy, 2010-2020.
